# C3 glomerulopathy in cystic fibrosis: a case report

**DOI:** 10.1186/s12882-018-0880-y

**Published:** 2018-03-28

**Authors:** Domenico Santoro, Rossella Siligato, Carmela Vadalà, Mariacristina Lucanto, Simona Cristadoro, Giovanni Conti, Michele Buemi, Stefano Costa, Ettore Sabadini, Giuseppe Magazzù

**Affiliations:** 1 Department of Clinical and Experimental Medicine, University of Messina, Via Faranda, 2-98123 Messina, Italy; 2 Unit of Pediatric Gastroenterology and Cystic Fibrosis, University of Messina, Messina, Italy; 3 Unit of Pediatric Nephrology and Rheumatology, University of Messina, Messina, Italy; 4 Unit of Nephrology, Hospital Riuniti, Bergamo, Italy

**Keywords:** C3 glomerulopathy, Renal biopsy, Cystic fibrosis, Inflammation

## Abstract

**Background:**

C3 glomerulonephritis is a rare glomerulopathy characterized at renal biopsy by C3 deposition, alone or with scanty immunoglobulins, as well as by an electron-dense material in mesangium, subendothelial and subepithelial space. An abnormal systemic activation of the alternative pathway of the complement cascade is responsible for the development of the disease if triggered by several possible environmental conditions. We report the first case in literature of a patient affected by cystic fibrosis and C3GN.

**Case presentation:**

Our case involves a young woman with cystic fibrosis, who had persistent microscopic hematuria, proteinuria and hypocomplementemia C3 for over three months. Renal biopsy confirmed the diagnosis of C3 glomerulopathy. Complement system dysregulation was tested and resulted in a strong terminal pathway activation proved by high levels of sC5b-9 complex, amounting to 1588 ng/ml (normal value < 400 ng/ml). Next generation sequencing (NGS) showed polymorphism in *CFH* (p.V62I in SCR1) and *THBD* (p.A473V), already known as pathogenic for C3GN, as well as a mutation in *C3* (p.R102G) associated only with age-related macular degeneration (AMD) so far. Treatment was based on ACE inhibitors and kidney function is currently stable (GFR 50 ml/min, serum creatinine 1.7).

**Conclusions:**

The co-existence of C3 glomerulopathy in a patient with CF, which is characterized by chronic infection/inflammation, makes this case an interesting model of chronic altered systemic activation of the alternative pathway of the complement cascade.

## Background

C3 glomerulonephritis (C3GN) has been recently included with Dense Deposit Disease (DDD) in the new entity of C3 glomerulopathy. This has been codified to highlight the difference between clearly recognizable secondary forms of membranoproliferative glomerulonephritis (MPGN) and those idiopathic cases lacking the classical staining for immunoglobulin at immunofluorescence (IF). The latter forms show, instead, an almost exclusive C3 staining- in some cases also C5b-9- without other complement factors belonging to either classical or lectin pathway, and they are associated to hypocomplementemia. At present time C3 glomerulopathy can be considered as a multifactorial disease, resulting from an altered control of the alternative pathway, triggered by outer conditions, such as infections that stimulate the already abnormal activation of complement [1].

C3 glomerulonephritis represents a very rare disorder with an incidence of 2–3 cases per million inhabitants per year. It affects equally both male and female population and the mean age of onset is 21 years old. Its onset is typically characterized by nephritic symptoms but it can also be associated with higher levels of proteinuria, even in nephrotic range. The natural course of this disease implies a 25% risk of ESRD progression in the following 10 years. Recurrence of C3GN after kidney transplant is observed in 60% of patients, usually in a mean range of 12–8 months after receiving the allograft [2].

Cystic fibrosis (CF) is the most common autosomal recessive disease affecting the Caucasian population, with a birth incidence ranging between 1:2500 and 1:4500 [3]. It is caused by mutations in *CFTR* (*cystic fibrosis transmembrane regulator*) gene which is localized on chromosome 7 (https://ghr.nlm.nih.gov/condition/cystic-fibrosis#statistics). Renal disease is reported as a relatively rare complication in adult patients with CF [4]. This is the first occurrence of C3 glomerulopathy in a patient with CF.

## Case presentation

Our case involved a 32 year-old woman affected by CF diagnosed at 6 months old, with renal diseases and hypertension in her family medical history. She also suffered from reactive arthritis since 19 years old in steroid and hydroxychloroquine therapy and had been diagnosed with CF-related diabetes at the age of 21.

After a fever episode (treated with ciprofloxacin) in October 2015 the patient’s laboratory tests showed Haemoglobin 8.2 mg/dl, serum creatinine 2 mg/dl, creatinine clearance 62 ml/min, hypocomplementemia C3. Her urinalysis pointed out hematuria (10–20 RBC/field of view) with 60% of dysmorphic erythrocytes, without proteinuria. The first clinical suspicion was a post-infectious glomerulonephritis. After that, a close follow up was conducted until December 2015. The persistent laboratory results over 8 weeks required kidney biopsy to be performed to exclude other causes of nephritic syndrome.

**Fig. 1:**
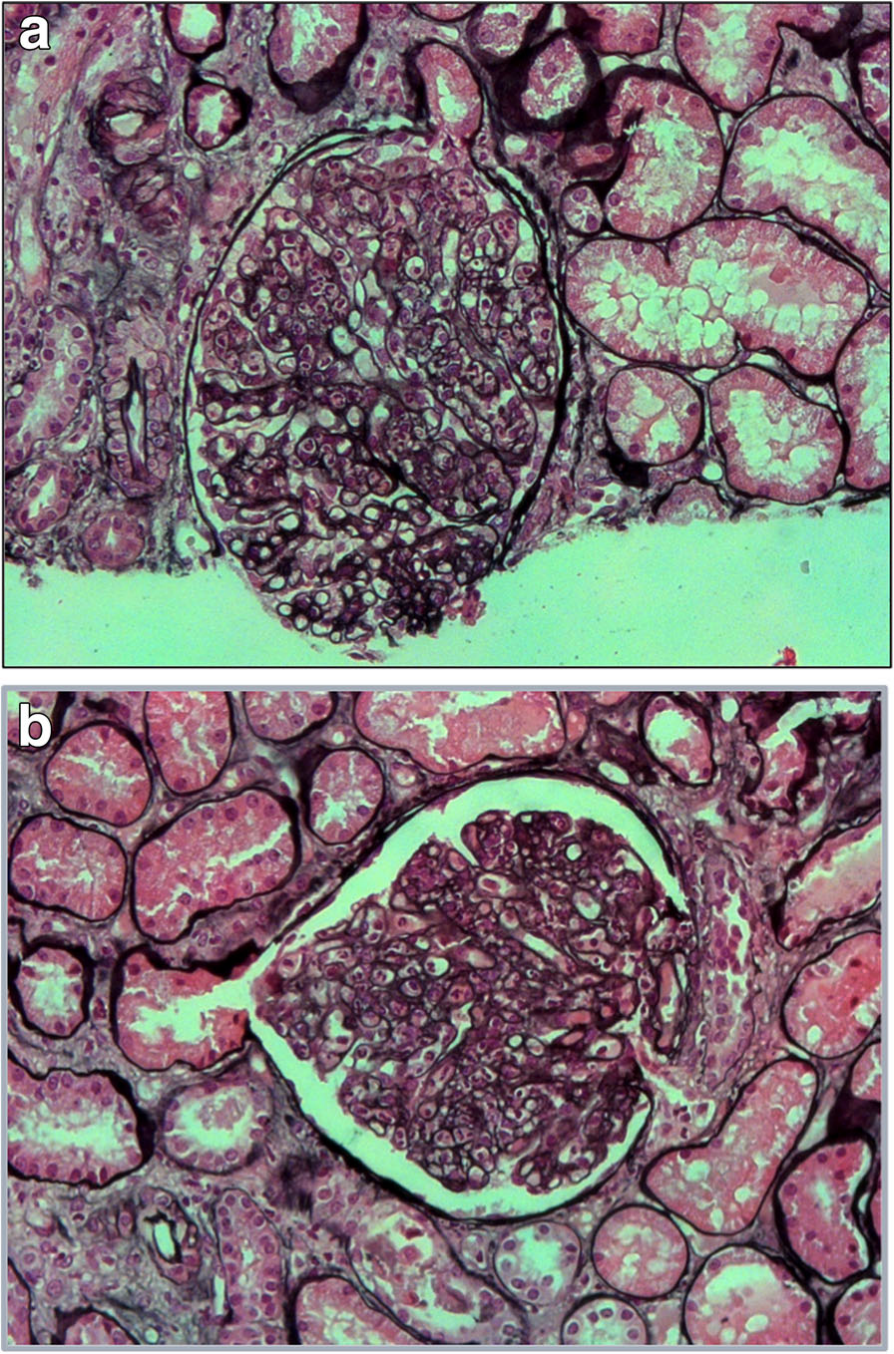
**a** and **b**. Light microscopy (Silver staining 40×): Endocapillary proliferation with neutrophil (and less frequently lympho-monocyte) infiltration

Light microscopy pointed out 5 sclerotic glomeruli out of 23. Main pattern was diffuse endocapillary proliferation, segmental in only a few glomeruli, with neutrophil (and less frequently lympho-monocyte) infiltration. Mesangium was characterized by deposits as well as hypercellularity and increased mesangial matrix. Interstitium showed mild edema and inflammatory cells, as well as between Tubular Basement Membrane and the above cells. A few arteries presented a fibrous intimal thickening. Arteriolar hyalinosis manifested too (Fig. 1a and b).

**Fig. 2:**
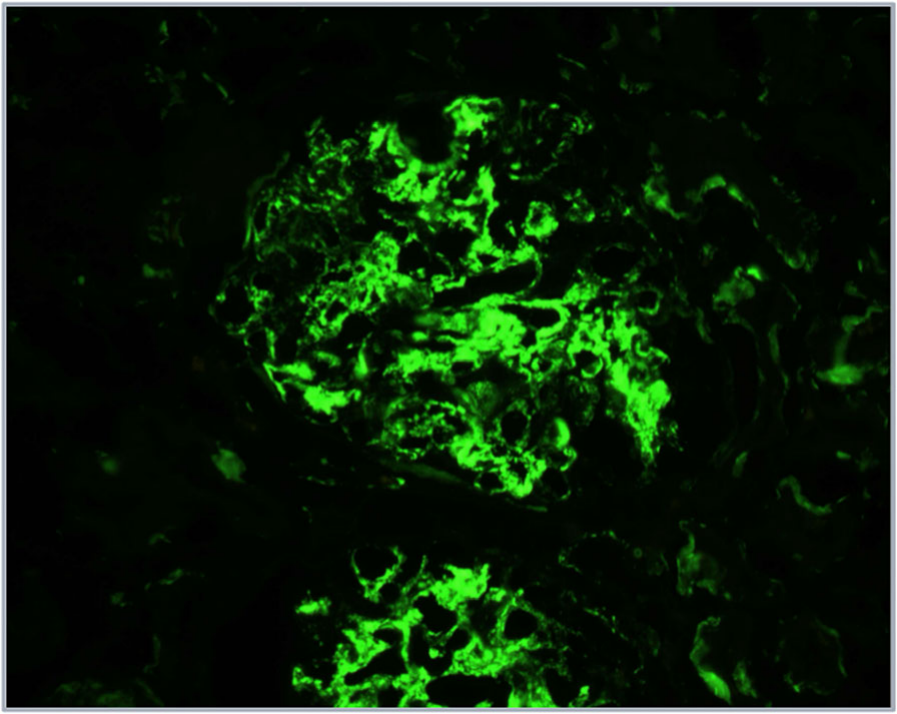
Immunofluorescence for C3: strong staining (3+) in granular deposits in mesangium and glomerular basement membrane (GBM). staining was less evident in tubuli (2+)

Immunofluorescence assay proved strong C3 staining (3+) in granular deposits in mesangium and glomerular basement membrane (GBM). Staining was less evident in tubuli (2+). IgA staining was focal in mesangium and in only a few arteriolar walls (2+). The presence of IgG only into tubular cells was explained as the result of re-absorption (Fig. 2).

**Fig. 3:**
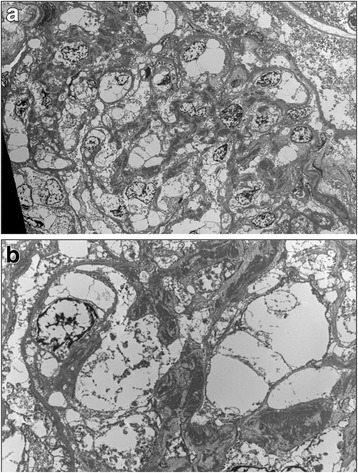
**a** and **b**. Electron microscopy Electron dense deposits were localized to a greater extent in mesangium, but also in subendothelial and subepithelial spaces

The deposits were well characterized by electron microscopy. They were localized to a greater extent in mesangium, but the assay demonstrated their presence also in subendothelial and subepithelial spaces. Their confluent electrondense appearance, instead of the intensely osmiophilic ribbon-shaped deposits pathognomonic for DDD, allowed to conclude for a C3 glomerulonephritis (Fig. 3a and b).

Complement system dysregulation was tested and resulted in a strong terminal pathway activation proved by high levels of sC5b-9 complex, amounting to 1588 ng/ml (normal value < 400 ng/ml).

Both genetics and C3NeF were investigated to determine the etiology of this altered control of alternative pathway. Next generation sequencing (NGS) showed polymorphism in *CFH* (p.V62I in SCR1) and *THBD* (p.A473V) already known as pathogenic for C3GN, as well as a mutation in *C3* (p.R102G) associated only with age-related macular degeneration (AMD) so far.^4^

Our patient is now treated with Angiotensin-converting-enzyme inhibitors (ACEI) added to her current Cystic Fybrosis and Reactive Arthritis therapies to preserve her kidney function.

After two years of disease her renal function is stable with serum creatinine 1.7 mg/dl, creatinine clearance 50 ml/min, 24 h proteinuria 270 mg, hypocomplementemia for C3 (14 mg/dl) and haemoglobin 9.4 mg/dl.

## Discussion

The patient is affected by two rare diseases, Cystic Fibrosis and C3 glomerulonephritis, whose association is not known in literature because of their different gene mutations and the lacking of a common pathogenesis.

First thing first we have to consider that the improved therapy of CF made possible a prolongation of life expectancy - from 20 years in 1960 beyond 40 years for patients born in 2000 [5] - that exposes the patients to the risk of developing age-related kidney disease. Moreover, this goal has been reached also with the prevention and treatment of infections with antibiotics, whose renal toxicity has to be carefully considered, given that it is cumulative especially in children and may cause chronic interstitial lesions in kidneys.

Secondly, we have to consider possible CF indirect kidney complications which are more evident with the longer estimated life of the patients. The progressive pancreatic failure and the development of diabetes may cause diabetic nephropathy. Equally, also chronic infections, which patients are more vulnerable to, may be responsible of AA amyloidosis that usually involves kidney with proteinuria, nephrotic syndrome and progressive renal insufficiency leading to ESRD.

In this case, besides the genetic predisposition to dysregulation of alternative complement pathway expressed by patient’s CFH, THBD and C3 polymorphism, one hypothesis regarding pathophysiology could be related to chronic infections due to CF. Indeed it may act as a trigger for the onset of C3 glomerulopathy stimulating a continuous activation of complement cascade, but we do not exclude other possible causes.

Complement dysregulation is the known cause of C3 glomerulopathy and can be congenital or acquired. Genetic mutations involve mainly fluid phase regulator factors of the alternative pathway such as *CFH* in its short consensus repeats (SCR) 1, 2, 6, 10 and 20. However, also *CFI* and *MCP* mutations were found [6, 7]. In particular, Martínez-Barricarte and coll [8] and Gale and coll [9] studied familiar forms of C3GN and found an association with a *CFHR3–1* hybrid protein, [10] an internal duplication of *CFHR1* [11] and another duplication interesting *CFHR5,* all genes codifying for proteins that share a high degree of homology with CFH [12]. All these mutations cause a loss of function that implies an abnormal activation of the alternative pathway.

An acquired mechanism of dysregulation involves C3 Nephritic Factor (C3NeF), an autoantibody that binds a neoepitope on the C3 convertase of the alternative pathway, stabilizing it against CFH-mediated decay and prolonging its C3 cleaving action. A second kind of C3NeF stabilizes the C3 convertase of the classical pathway, incrementing C3 and C5 activation but is properdin-dependent. C3NeF can be found in up to 50% of patients with C3 glomerulopathy, with lower titer in C3GN compared to DDD patients.

Also factor H autoantibodies (FHAA) binding SCR 3 and altering FH interaction with C3b have been identified, although they are rare in C3GN and their effective pathogenic role has to be investigated yet [6].

In our patient we found two polymorphisms already known as pathogenic for C3GN in *CFH* (p.V62I in SCR1) and *THBD* (p.A473V) genes, as well as a polymorphism so far associated to age-related macular degeneration (AMD) in *C3* (p.R102G) [13]. These alterations may express a certain degree of predisposition to the dysregulation of alternative complement pathway.

Our patient is now treated with ACEI since no other therapies have been demonstrated to be effective in the treatment of this disorder. ACEI and Angiotensin II receptor blocker (ARB) are given to most of the patients because of their antiproteinuric and antihypertensive effect, thus playing a nephroprotective role in both nephritic and nephrotic presentation. This strategy has been borrowed in other glomerulonephritis and it is not specific for this disease. Our patient is in lung transplant waiting list at the moment.

Several other approaches are currently used for C3 glomerulopathy lacking a gold standard treatment. Long term steroid therapy alone or in combined approach with mycophenolate efficacy is yet to be proven because the only study which demonstrated beneficial effects on kidney function regarded MPGN cases before of their distinction in immunocomplex mediated and C3 glomerulopathy [14, 15].

Plasma therapy, in its variants of plasma exchange (PE) and plasma infusion (PI), has been adopted in selected cases of DDD with mutated *CFH,* but in only a few reports demonstrated its effectiveness [16].

Evidence of C3NeF and occasionally of FHAA addressed attention to immunosoppressor targeted therapies such as rituximab and eculizumab, respectively humanized anti-CD20 and anti-C5 monoclonal antibodies. Eculizumab in particular has been designed first to treat paroxysmal nocturnal hemoglobinuria (PNH) and atypical hemolitic uremic syndrome (aHUS). Because of the common genetic background to aHUS and C3 glomerulopathy, this drug is now an off-label treatment administered to few patients and with variable effectiveness. Inactivation of C5 eculizumab mediated in a *CFH* deficient mouse model proved that the C3GN phenotype can develop with regular C3 deposition and endothelial alterations as well as proteinuria, but with less glomerular inflammation [17]. In our case, the hypothesis of a therapy with eculizumab was initially considered. Indeed, data seem to prove that the best degree of efficacy can be reached treating with eculizumab those patients who have high levels of sC5b-9 complex that lead to cell apoptosis. For the moment this drug has been used in anecdotal cases and in a single non-randomized trial for the treatment of C3 glomerulopathies [18]. However, in this study patients were selected on the basis of a high risk of progression, defined by a proteinuria of more than 1 g / day or acute renal failure [18]. In our case, stable renal function and low degree of proteinuria, as well as the increased risk of infections, to which the inhibition of the complement system would inevitably expose, did not represent indications to eculizumab therapy.

Finally, it is worth considering the relationship between kidney function and lung transplant. This is an advanced stage therapy for CF and requires a careful monitoring of renal function for an appropriate management of both pre- and post-operative periods, because of the surgery impact as well as the subsequent huge use of antibiotics and immunosuppressor drugs, with high risk of nephrotoxicity. An analysis led by Lefaucheur and coll. Registered a faster reduction of glomerular filtration rate (GFR) in 32.5% of transplanted lung patients and a strong association with the risk of developing ESRD [19].

## Conclusions

We reported for the first time the occurrence of C3 glomerulopathy in CF. We speculate that CF may act as a trigger for continuous activation of complement system due to a predisposing condition of alternative pathway dysregulation.

## Abbreviations

ACEI: Angiotensin-converting-enzyme inhibitors
aHUS: atypical hemolitic uremic syndrome
AMD: Age-related macular degeneration
ARB: Angiotensin II receptor blocker
C3GN: C3 glomerulonephritis
C3NeF: C3 Nephritic factor
CF: Cystic fibrosis
*CFTR*: *Cystic fibrosis transmembrane regulator*
DDD: Dense Deposit Disease
ESRD: End stage renal disease
FHAA: Factor H autoantibodies
GFR: Glomerular filtration rate
MPGN: Membranoproliferative glomerulonephritis
PE: Plasma exchange
PI: Plasma infusion
PNH: Paroxysmal nocturnal hemoglobinuria
SCR: Short consensus repeats
